# Supplementary material for: Allosteric regulation and crystallographic fragment screening of SARS-CoV-2 NSP15 endoribonuclease
Source: Nucleic Acids Res. 2023 Apr 28;51(10):5255–70. doi: 10.1093/nar/gkad314 (PMC10250223; doi:10.1093/nar/gkad314)
Supplement: gkad314_Supplemental_Files [file gkad314_supplemental_files.zip › Supplementary datasets.zip!/224/Refine_9/01-BUSTER/Cycle-1/LIST.html]

Refining

**BUSTER 2.X**: A suite of programs for macromolecular structure refinement by maximum-likelihood and Bayesian methods  
Copyright © 1992-2020 Global Phasing Ltd.  
All rights reserved.  
Authors: G. Bricogne, E. Blanc, M. Brandl, C. Flensburg, P. Keller, W. Paciorek, P. Roversi, O. Smart, C. Vonrhein, T. Womack  

Please reference use of the software as:

- BUSTER-TNT 2.X, Global Phasing Ltd, Sheraton House, Cambridge CB3 0AX, UK

Version: 2.10.3 for linux64-ifort made on May 20 2020 at 01:14:34  
Licenced to: Diamond Light Source, Scientific Software  

```
---


### Parsing of the BUSTER cardfile

TITLE : autoBUSTER refinement

   Cell information

   Symmetry information

 Using CCP4 X-ray form factors.

   Missing structure chemical composition

 Using ATOM block for chemical composition.


   Shell information

### Parsing of the TNT cardfile


---


### Reading observations file

 Number of reflexions in input:  58343

      Number of accepted reflexions:  52749

      Number of rejected reflexions:      0

   Reflexions in test set:            2702

   Reflexions in working set:        50047


---


### 1. Generating initial model

 2.10 * Shannon sampling rate. NX NY NZ:   288  288  240


  Effective Shannon rates are:    2.29    2.29    2.55


   Allocated BUFFER of size:     76.465 Mb.

   Initial Geometry Information


 No missing atoms declared


   Summary of model volumes

 No missing atoms declared: no prior computed


 Computing bulk solvent model scattering


 NCSMASK PDB radius for solvent mask:     2.15 Angstrom

 running PDBSET (setting cell and spacegroup P1) ... done
 running NCSMASK [1] (creating mask, SMOOTH 1.04842) ... done
 running MAPDUMP (to check if reduction to unit cell is needed) ... done
 running MAPMASK (converting mask to map) ... done

   Generation of babslv by theta-filter

   Electron density in frg mask:  0.420 Å-1

 FOBS  put on rough absolute scale by Wilson plot :
           K        =  0.65964E+02
           B        =    -12.578
   Setting starting scale K to value from Wilson scaling:  65.96432
   Setting starting scale B to value from Wilson scaling: -12.57769

 

### Cycle 0001: MaxLik scaling and refinement


   MaxLik Scaling Cycle 0001

   Wilson plot

   Statistics in resolution bins just after scaling
```

|  |  |
| --- | --- |
|  | - Rxpct factors plot- Rxpct factors table- <Log-Lik Gain>- Ln Sigma A- Correlation Coefficients |

```
   <Log-Likelihood> of null-hypothesis

Structure Factor Amplitudes


   Average Figure of Merit

 Switching off refinement of overall temperature factor


 Added B =     8.7596 Å2 to all atoms in fragment


 Added B =     8.7596 Å2 to B_SOLV

```
gelly refinement module
 Authors (2004-2009) O. S. Smart, C. Flensburg, W. Paciorek, C. Vonrhein, 
                     M. Brandl, T. O. Womack and G. Bricogne
         (2010-2011) O. S. Smart, C. Flensburg, W. Paciorek, C. Vonrhein, 
                     T. O. Womack and G. Bricogne
         (2012-2014) O. S. Smart, C. Flensburg, W. Paciorek, C. Vonrhein, 
                     and G. Bricogne
         (2015-2020) G. Bricogne, C. Flensburg, W. Paciorek, C. Vonrhein
 Copyright © 2004-2020 Global Phasing Ltd. 
 All rights reserved.
 gelly module compilation date May 20 2020 01:11:20
 
 gelly uses routines from TNT geometry. References: 
     Tronrud, D. E., Ten Eyck, L. F., Matthews, B. W. (1987)
     "An Efficient General-Purpose Least-Squares Refinement Program 
     for Macromolecular Structures". Acta Crystallogr A, 43:489-501.
     Tronrud, D. E. (1997). "The TNT Refinement Package", 
     in Macromolecular Crystallography, Part B, Eds Charlie Carter 
     and Robert Sweet, Methods in Enzymology, 277:306-319.
 wildcard matching function by Julian Robichaux http://www.nsftools.com

 No -random_seed flag found. Random number stream initialised using:
2147483562
2147483398

 Control flags set to:  -autoncs -hydrogens_input_occ2zero \
                     -hydrogens_output_occ2heavy -screen \
                     100 -screen_sigma \
                     3.0 -keeppdb \
                     1000000 -taomonitor_verbose \
                     2 
 

 ############### Starting setup via TNT routines

 Setting up tnt routines including license check:

                   TNT Stereochemistry Program
 Version: 5.22.3.0
 Licence for Diamond Light Source, Scientific Software is valid
 
 Using TNT geometry routines to load the tnt control file: gelly.tnt
 Using TNT routines to load initial coordinates from file fragment.cor
 Expecting 6506 atoms

 Found     2197 "NOTE BUSTER_RESIDUE_ATOM_DATA" cards
 Have copied information for    6506 atoms from TNT to gelly's store 
 
 The TNT WEIGHT cards used by this run:
 -----WEIGHT       BOND     2.0
 -----WEIGHT       ANGLE    2.0
 -----WEIGHT       IMPROPER 0.0
 -----WEIGHT       TORSION  2.0
 -----WEIGHT       PSEUDO   0.0
 -----WEIGHT       TRIGONAL 2.0
 -----WEIGHT       PLANE    5.0
 -----WEIGHT       CONTACT  5.0
 -----WEIGHT       BCORREL  10.0
 -----WEIGHT       CHIRAL   5.0
 -----WEIGHT       NCS      50.0
 -----WEIGHT       IDEAL    4.0
 -----WEIGHT       RFACTOR  4
 
 Dictionary versions picked up from NOTE BUSTER_RESTRAINT_DICT cards as: 
 ------ protgeo_eh99.dat (V1.6.4.17) 20191001  STANDARD AMINO ACID DICTIONARY. BONDS AND ANGLES FROM ENGH AND HUBER EH99. OTHER VALUES BASED ON PREVIOUS TNT OR TAKEN FROM CCP4. INCLUDES HYDROGEN ATOMS.
 ------ exoticaa.dat (V1.3.4.15) 20200327  COLLECTION OF NON-STANDARD AMINO ACIDS, MAINLY EH91 WITHOUT IDEAL DISTANCE INFO
 ------ nuclgeo.dat (V1.13.4.8) 20190909
 ------ bcorrel.dat (V1.15.10.2) 20190616
 ------ contact.dat (V1.15.12.10) 20191121
 ------ idealdist_contact.dat (V1.3.4.12) 20191001  IDEAL-DISTANCE CONTACT TERM DATA AS USED IN PROLSQ. VALUES USED HERE ARE BASED ON DATA HARVESTING OF PDB AS OF 20190109.
 ------ restraints for LIG (Unknown) from cif dictionary Z927400026.cif
 ------ restraints for CIT (CITRIC ACID) from cif dictionary CIT.cif; generated by GRADE_PDB_LIGAND 1.2.12 (October 4 2016) using MOGUL 1.7.2(RC1), CSD as538be, with quantum mechanics RM1 ((DatabaseID PDB-CIT))
 ------ assume.dat (V1.9.4.2) 20180705
 
 Have picked up spacegroup name for pdb output as 'P 63      '
 Setting up Chain/Residue arrays
 
 Have command line specification -autoncs*
     Have found       1 NCS relations
     Pairwise matching residue number and type between chains: 
                    A        B        S        W    
          A         .       99%       -        -   
          B                  .        -        -   
          S                           .        -   
          W                                    .   
       Table key--                                                       
          all   : two chains have 100% match and same number of residues 
         multi  : ditto and they contribute to a multi chain ncs cluster 
          ***%  : two chains have >80% matching residue number/type      
           -    : chains appear to be unrelated                          
      chain A     have identified ncs to     1 other chains. So will weight each autoncs by (       6.666/#)=      6.6660
      chain B     have identified ncs to     1 other chains. So will weight each autoncs by (       6.666/#)=      6.6660
      chain S     have identified ncs to     0 other chains. 
      chain W     have identified ncs to     0 other chains. 
     For .Gelly cards produced see below - after pruning
 
 Setting up BUSTER_SET groups
 Have found    93 BUSTER_SET cards
 Now silently applying automatic/dictionary set definitions. For a verbose listing use '-verbose_set' flag.
 Applying user defined set definitions:
 =====NOTE BUSTER_SET TLSatoms = Empty --> set 'TLSatoms' now has     0 atoms 
 =====NOTE BUSTER_SET TLSatoms = TLSatoms + { A|* } --> set 'TLSatoms' now has  2770 atoms 
 =====NOTE BUSTER_SET TLSatoms = TLSatoms + { B|* } --> set 'TLSatoms' now has  5540 atoms 
 =====NOTE BUSTER_SET notTLSatoms = \ TLSatoms --> set 'notTLSatoms' now has   966 atoms 
 =====NOTE BUSTER_SET ncsautoXcld = Water --> set 'ncsautoXcld' now has   966 atoms 
 =====NOTE BUSTER_SET ncsautoABset = Chain_A \ ncsautoXcld --> set 'ncsautoABset' now has  2770 atoms 
 Have defined    51 sets: 
      set:                 'All' has   6506 atoms in   1666 residues - automatically defined
      set:               'Empty' has      0 atoms in      0 residues - automatically defined
      set:            'ExoticAA' has      0 atoms in      0 residues - defined by dictionary file
      set:           'ExoticNUC' has      0 atoms in      0 residues - defined by dictionary file
      set:              'NotHET' has   5514 atoms in    698 residues - defined by dictionary file
      set:             'Chain_A' has   2770 atoms in    350 residues - automatically defined
      set:             'Chain_B' has   2770 atoms in    350 residues - automatically defined
      set:             'Chain_S' has    567 atoms in    567 residues - automatically defined
      set:             'Chain_W' has    399 atoms in    399 residues - automatically defined
      set:               'Water' has    966 atoms in    966 residues - defined by dictionary file
      set:          'StdProtein' has   5514 atoms in    698 residues - defined by dictionary file
      set:             'Protein' has   5514 atoms in    698 residues - defined by dictionary file
      set:                'Back' has   2794 atoms in    698 residues - defined by dictionary file
      set:                'Side' has   2720 atoms in    650 residues - defined by dictionary file
      set:        'RnaCanonical' has      0 atoms in      0 residues - defined by dictionary file
      set:        'DnaCanonical' has      0 atoms in      0 residues - defined by dictionary file
      set:     'RnaDnaCanonical' has      0 atoms in      0 residues - defined by dictionary file
      set:     'DnaRnaCanonical' has      0 atoms in      0 residues - defined by dictionary file
      set:         'RnaExtended' has      0 atoms in      0 residues - defined by dictionary file
      set:         'DnaExtended' has      0 atoms in      0 residues - defined by dictionary file
      set:      'RnaDnaExtended' has      0 atoms in      0 residues - defined by dictionary file
      set:      'DnaRnaExtended' has      0 atoms in      0 residues - defined by dictionary file
      set:                 'Dna' has      0 atoms in      0 residues - defined by dictionary file
      set:                 'Rna' has      0 atoms in      0 residues - defined by dictionary file
      set:              'RnaDna' has      0 atoms in      0 residues - defined by dictionary file
      set:              'DnaRna' has      0 atoms in      0 residues - defined by dictionary file
      set:             'Nucleic' has      0 atoms in      0 residues - defined by dictionary file
      set:             'Polymer' has   5514 atoms in    698 residues - defined by dictionary file
      set: 'BufferWeichenberger' has     13 atoms in      1 residues - defined by dictionary file
      set:    'BufferAdditional' has      0 atoms in      0 residues - defined by dictionary file
      set:              'Buffer' has     13 atoms in      1 residues - defined by dictionary file
      set:         'MetalAlkali' has      0 atoms in      0 residues - defined by dictionary file
      set:  'MetalAlkalineEarth' has      0 atoms in      0 residues - defined by dictionary file
      set:     'MetalTransition' has      0 atoms in      0 residues - defined by dictionary file
      set: 'MetalPostTransition' has      0 atoms in      0 residues - defined by dictionary file
      set:    'MetalLanthanides' has      0 atoms in      0 residues - defined by dictionary file
      set:               'Metal' has      0 atoms in      0 residues - defined by dictionary file
      set:         'SugarAlphaD' has      0 atoms in      0 residues - defined by dictionary file
      set:         'SugarAlphaL' has      0 atoms in      0 residues - defined by dictionary file
      set:               'Sugar' has      0 atoms in      0 residues - defined by dictionary file
      set:               'Other' has     26 atoms in      2 residues - defined by dictionary file
      set:            'Hydrogen' has      0 atoms in      0 residues - defined by dictionary file
      set:         'nonHydrogen' has   6506 atoms in   1666 residues - defined by dictionary file
      set:             'OccZero' has      0 atoms in      0 residues - defined by dictionary file
      set:          'OccNonZero' has   6506 atoms in   1666 residues - defined by dictionary file
      set:             'OccFull' has   6461 atoms in   1663 residues - defined by dictionary file
      set:          'OccNonFull' has     45 atoms in      3 residues - defined by dictionary file
      set:            'TLSatoms' has   5540 atoms in    700 residues - user specified
      set:         'notTLSatoms' has    966 atoms in    966 residues - user specified
      set:         'ncsautoXcld' has    966 atoms in    966 residues - user specified
      set:        'ncsautoABset' has   2770 atoms in    350 residues - user specified
 No command line arguments '-occ_allhet' or '-occ_restyp' so will not setup automated occcupancy refinement
 
 Interpreting CONSTANT and FREE cards: 
 =====CONSTANT     OCC
 =====NOTE BUSTER_CONSTANT B   TLSatoms
 Have found and interpreted      2 CONSTANT cards in the TNT input. 
 Before any COMBINE - Number of atoms with fixed XYZ =     0
 Before any COMBINE - Number of atoms with fixed B   =  5540
 Before any COMBINE - Number of atoms with fixed OCC =  6506
 Before any COMBINE - Number of atoms with fixed BIJ =   966
                            cf total number of atoms =  6506
 
 There are no COMBINE cards to be found in the TNT card input 
 
 There are no NOTE BUSTER_COMBINE_RES cards to be found in the TNT card input 

 ############### Working out full connectivity list


 ############### Working out equivalent atoms


 Residue with most atoms given below:
        A|58:N(TRP)         
        A|58:CA(TRP)        
        A|58:CB(TRP)        
        A|58:CG(TRP)        
        A|58:CD1(TRP)       
        A|58:NE1(TRP)       
        A|58:CE2(TRP)       
        A|58:CD2(TRP)       
        A|58:CE3(TRP)       
        A|58:CZ3(TRP)       
        A|58:CH2(TRP)       
        A|58:CZ2(TRP)       
        A|58:C(TRP)         
        A|58:O(TRP)         


 Have no '-equiv_check_plus' argument - so will take atom type into
 account when checking for equivalence of otherwise identical atoms.
 

 ############### Looking at dihedrals


 ############### Looking at chirals

 NOTE BUSTER_CHIRAL_ANGLECUT card found - read value=    -31.00 degs from it
 NOTE BUSTER_CHIRAL_SIGMA card found - read value=      1.70 degs from it
 RANGE allowed for B variables in refinement:   from      3.00000 to    300.00000

 ############### Preparing for PDB output


 ############### Processing extra restraints explicitly defined

 No 'NOTE BUSTER_TARGET' cards or -target?? args found (used to read coordinates files for soft ncs to external)
 No 'NOTE BUSTER_OCCSUM'     cards found - so not using sum of occupancy restraints
 No 'NOTE BUSTER_DISTANCE'   cards found - so not setting up any additional atom-atom distance restraints
 No 'NOTE BUSTER_UTILANGLE'  cards found - so not setting up any additional restraints on any angle
 No 'NOTE BUSTER_UTILTOR'    cards found - so not setting up any additional sinusoidal restraints on torsions
 
 --- Have some similarity definitions 
     Similarity restraints on RMSD use superposition routines from: 
     Coutsias, E.A., Seok, C., Dill, K.A.(2004)
     "Using quaternions to calculate RMSD", J. Comput. Chem., 25:1849-1857 

 Have found    1 'NOTE BUSTER_SIM_DEFINE' cards 
     Maximum number of chains involved in any SIM group (excluding the template)=     1


 ########## Interpreting card: NOTE BUSTER_SIM_DEFINE ncsautoAB ncsautoABset B
     Number of atoms in template set=  2770
     Number of related chains (other than template) in SIM set=    1
     List of chains to be restrained (excluding template)=   B   
     Pairwise RMSD, XYZ in Angstroms, for SIM group: ncsautoAB
                 A         B   
     A         -----     ----- 
     B         0.653     ----- 
     Overall RMSD,XYZ for the SIM group is     0.653 Angstroms
     Pairwise rms deviation of [individual B's minus their set/chain average] in Angs**2
                 A         B         Bavg
     A         -----     -----     86.91
     B         27.73     -----     68.03
     Overall RMSD,B   for the SIM group is    27.725 Angs**2
     The number of common atoms for the above fits:
                 A         B   
     A         -----     ----- 
     B          2757     ----- 
     (compared to the template set that has    2770 atoms in it)
     List of atoms that are absent in one or more chains for this group: 
                                    A    B   
                    C1      401     +    -
                    O1      401     +    -
                    O2      401     +    -
                    C2      401     +    -
                    C3      401     +    -
                    O7      401     +    -
                    C4      401     +    -
                    C5      401     +    -
                    O3      401     +    -
                    O4      401     +    -
                    C6      401     +    -
                    O5      401     +    -
                    O6      401     +    -
     (key  +  atom present for this chain 
           -  atom absent  for this chain)
 transforms for each of the superpositions orthogonal matrix r11 r12 r13 r21 r22 r23 r31 r32 r33 tx ty tz (PDBSET syntax): 
TRANS_AB -0.877148 -0.480058 -0.012516 -0.480080  0.877224 -0.001364  0.011634  0.004812 -0.999921    33.905    33.021    15.299
 reverse transforms 
TRANS_BA -0.877148 -0.480080  0.011634 -0.480058  0.877224  0.004812 -0.012516 -0.001364 -0.999921    45.414   -12.765    15.767
 
 Setting up LSSR interatomic distance restraints
 Interpreting card: NOTE BUSTER_SIM_RESTRAIN_LSSR ncsautoAB 6.666
        setting up interatomic distance pair restraints with:
        Dmin=         0.00
        Dmax=         5.50
        sigma=        0.20
        Vmax=         3.00
        weight=       6.67
        number of distance pairs defined for this set is      35121
 Have not supplied either '-sim_swap_equiv' or '-sim_swap_equiv_plus' argument 
   so will not try swaping equivalent atoms to improve NCS and/or target agreement.
 
 NCS by LSSR outlier analysis
 -autoncs option automatic NCS prune routine -------- 
 Residues pruned out by gradient condition:
          A|253     B|253
  
 Residues pruned out by function/max condition:
          A|149     A|150     A|173     A|187     A|188     A|216     A|220 
          A|259     A|346    A|ENDA     B|149     B|150     B|173     B|187 
          B|188     B|216     B|220     B|259     B|346    B|ENDA 
  
 Total number of residues for which LSSR restraints have been pruned out is       22 out of      698 participating
 Number of LSSR restraints pruned out is      1456 this is    4.1% of the total           35121
  
 -autoncs .Gelly cards suitable for adaptation for manual definition/pruning
           (note auto pruning is done in a slightly different way so exact results may differ slightly)
 -autoncs option .Gelly cards also written to file  auto_gelly_cards.txt
NOTE BUSTER_SET ncsautoXcld = Water    # card added - produced by -autoncs
NOTE BUSTER_SET ncsautoXcld = ncsautoXcld + { A|149 A|150 A|173 A|187 A|188 }    # card added - produced by -autoncs
NOTE BUSTER_SET ncsautoXcld = ncsautoXcld + { A|216 A|220 A|253 A|259 A|346 }    # card added - produced by -autoncs
NOTE BUSTER_SET ncsautoXcld = ncsautoXcld + { A|ENDA }    # card added - produced by -autoncs
NOTE BUSTER_SET ncsautoABset = Chain_A \ ncsautoXcld    # card added - produced by -autoncs
NOTE BUSTER_SIM_DEFINE ncsautoAB ncsautoABset B    # card added - produced by -autoncs
NOTE BUSTER_SIM_RESTRAIN_LSSR ncsautoAB 6.666    # card added - produced by -autoncs

 LSSR function contribution before pruning    119648.3
                             after pruning    104483.8
 Graph of which residues pruned from LSSR: ncs_pruned.mtv
 Graph of LSSR function outliers ncs_function_outliers.mtv
 Graph of LSSR gradient outliers ncs_gradient_outliers.mtv
 LSSR - analysis of distance difference distribution for NCS
         DeltaD less than 0.5 Angs:  proportion of population= 96.80% average DeltaD of subset     0.096 Angs
 Histogram of LSSR deltaD distribution ncs_histogram.mtv
 
 Interpreting card: NOTE BUSTER_TLS_SET tls1 { A|* }
 Interpreting card: NOTE BUSTER_TLS_SET tls2 { B|* }
 Interpreting card: NOTE BUSTER_TLS_SET tls1 { A|* }
 Interpreting card: NOTE BUSTER_TLS_SET tls2 { B|* }
 Interpreting card: NOTE BUSTER_TLS_FIX tls1 RB
 Interpreting card: NOTE BUSTER_TLS_FIX tls2 RB


 TLS information. Total number of groups:     2


 Group   Natoms    FixRB   FixTLS   Group Tag    Group Spec
     1     2770      Y        N     tls1    { A|* }
     2     2770      Y        N     tls2    { B|* }
 
NOTE BUSTER_TLS_SET tls1 { A|* }
NOTE BUSTER_TLS_T   tls1      0.00000E+00  0.00000E+00  0.00000E+00  0.00000E+00  0.00000E+00  0.00000E+00
NOTE BUSTER_TLS_L   tls1      0.00000E+00  0.00000E+00  0.00000E+00  0.00000E+00  0.00000E+00  0.00000E+00
NOTE BUSTER_TLS_S   tls1      0.00000E+00  0.00000E+00  0.00000E+00  0.00000E+00  0.00000E+00  0.00000E+00  0.00000E+00  0.00000E+00
NOTE BUSTER_TLS_O   tls1     -2.34016E+01 -8.37990E+01  1.17779E+01
NOTE BUSTER_TLS_FIX tls1 RB
 
NOTE BUSTER_TLS_SET tls2 { B|* }
NOTE BUSTER_TLS_T   tls2      0.00000E+00  0.00000E+00  0.00000E+00  0.00000E+00  0.00000E+00  0.00000E+00
NOTE BUSTER_TLS_L   tls2      0.00000E+00  0.00000E+00  0.00000E+00  0.00000E+00  0.00000E+00  0.00000E+00
NOTE BUSTER_TLS_S   tls2      0.00000E+00  0.00000E+00  0.00000E+00  0.00000E+00  0.00000E+00  0.00000E+00  0.00000E+00  0.00000E+00
NOTE BUSTER_TLS_O   tls2      2.21707E+01 -7.20650E+01  3.37962E+00
NOTE BUSTER_TLS_FIX tls2 RB


 TLS: mmCIF group specification begin


#
loop_
_pdbx_refine_tls_group.id
_pdbx_refine_tls_group.refine_tls_id
_pdbx_refine_tls_group.beg_PDB_ins_code
_pdbx_refine_tls_group.beg_auth_asym_id
_pdbx_refine_tls_group.beg_auth_seq_id
_pdbx_refine_tls_group.end_PDB_ins_code
_pdbx_refine_tls_group.end_auth_asym_id
_pdbx_refine_tls_group.end_auth_seq_id
_pdbx_refine_tls_group.selection
_pdbx_refine_tls_group.selection_details
   1   1 . A   -1 . A  346 . '{ A|* }'
   2   1 . A  401 . A  401 . '{ A|* }'
   3   2 . B   -1 . B  346 . '{ B|* }'
   4   2 . B  401 . B  401 . '{ B|* }'
#


 TLS: mmCIF group specification end


 Interpreting card: NOTE BUSTER_ANISO_SET Empty
 Interpreting card: NOTE BUSTER_ANISO_WEIGHT SCA  16.0
 Interpreting card: NOTE BUSTER_ANISO_WEIGHT SPH   0.6    0.6
 Interpreting card: NOTE BUSTER_ANISO_WEIGHT SIM   7.8    4.8    2.6    0
 Interpreting card: NOTE BUSTER_ANISO_WEIGHT RIG  21.5   21.5    3.7    0


 After TLS and ANISO: Number of atoms with fixed/combined xyz =     0
 After TLS and ANISO: Number of atoms with fixed/combined B   =  5540
 After TLS and ANISO: Number of atoms with fixed/combined OCC =  6506
 After TLS and ANISO: Number of atoms with fixed/combined BIJ =  6506
 After TLS and ANISO: Number of atoms in a TLS group          =  5540
                                     cf total number of atoms =  6506
 

 ############### Setup state vector

 No free_vect_ulim_xyz argument found: using default of  100.000
 No free_vect_llim_xyz argument found: using default of -100.000
 Number of parameters added for TLS description                 =      40
 Number of parameters to describe molecular conformation        =    3904

 ############### Setup free vector

 Number of free parameters to be adjusted in refinement   =    3904

 ############### Setting up own geometry


 No command line argument '-tnt_fn' so will use gelly to do geometry function calculations

 ============= Call TNT functions


 ============= Get TNT symmetry information

 
      Getting symmetry operators from TNT. 
          gelly will classify symmetry using pdb-like convention: 
               SYMOP   SYMMETRY
              NNNMMM   OPERATOR
                1555   X,Y,Z
                2555   -Y,X-Y,Z
                3555   -X+Y,-X,Z
                4555   -X,-Y,1/2+Z
                5555   Y,-X+Y,1/2+Z
                6555   X-Y,X,1/2+Z
          where NNN -> operator number and MMM -> translation vector
 

 ============= Own Gelly geometry/restraints setup


 ----------- Identify bond-types


    Setting up GEOMBONDREC_TYPE_HASH* to be able to map a given bond type (e.g.
    "single") to an internal integer (GBOND_TYPE=1) etc.

    Using default  GEOMBONDREC_TYPE_HASH_STR='SING* 1 DOUB* 2 TRIP* 3 AROM* 4 DELO* 4 METAL* 5':
    to override default use a NOTE BUSTER_GEOMBONDREC_TYPE_HASH_STR gelly card.

 ----------- Classify torsion into peptide omega and other


 ----------- Setup of non-bonded terms

 Ideal-distance contact term setup:
    Restraint function constants (normally these values are from NOTE BUSTER_IDEAL_CONTACT_* cards)
    Sigma for general contacts      0.200 Angs
    Sigma for 1-4 contacts          0.100 Angs (contacts across a torsion)
    Sigma for 1-5 contacts          0.150 Angs
    Sigma for 1-N contacts          0.200 Angs
    Sigma for HBOND pairs           0.100 Angs (contacts between HB_types A and D or B's)
    Contact distance increment for each atom in HBOND          -0.150 Angs 
    Contact distance increment for H-bond acceptor              0.100 Angs 
    Contact distance increment for 1-4 with an O atom          -0.050 Angs 
    Contact distance increment for 1-4 with an N atom          -0.180 Angs 
    Contact distance increment for 1-4 with an C atom          -0.370 Angs 
    Contact distance increment for 1-4 with an S atom          -0.150 Angs 
    Contact distance increment for 1-4 with an P atom          -0.150 Angs 
    Contact distance increment for 1-4 with any other atom     -0.150 Angs 
    All atoms matched for CCP4 atom type.
    Number of CCP4 atom types used is         25 (H-bound and not H-bound counted separately)

 Setting up short/ideal-distance contacts
   Using gelly special position handling. (For TNT instead rerun with argument '-special_tnt')

   No command line argument '-special_all' specified. We will auto-generate
   EXCLUDE cards only for atom-atom contacts across symmetry (i.e. on special
   position) when this involves a contact within a residue to itself.

   Have set up non-bonded exclusions - 1-3 contacts
   Atom A|27:CA         makes the most 1-3 contacts:  6

   Have set up non-bonded exclusions - 1-4 contacts
   Atom A|27:N          makes the most 1-4 contacts:  7

   Have set up non-bonded exclusions - 1-5 contacts
   Atom A|28:C.A        makes the most 1-5 contacts: 10

 ############################################################################################################
 ###################################################### setting up contact terms
 ############################################################################################################


 ============= Call Gelly versions of TNT functions

 WARNING: 1-4 contact without 1-(2,3)-4 data for A|401:O2(CIT)        and A|401:C3(CIT)        X-X
 WARNING: 1-4 contact without 1-(2,3)-4 data for A|401:C2(CIT)        and A|401:O6(CIT)        X-X
 WARNING: 1-4 contact without 1-(2,3)-4 data for B|401:C01(LIG)       and B|401:N05(LIG)       X-X
 WARNING: 1-4 contact without 1-(2,3)-4 data for B|401:N08(LIG)       and B|401:O11(LIG)       X-X
 WARNING: 1-4 contact without 1-(2,3)-4 data for B|401:C09(LIG)       and B|401:C12(LIG)       X-X
 WARNING: 1-4 contact without 1-(2,3)-4 data for B|401:C10(LIG)       and B|401:C13(LIG)       X-X

       TERM      weight      number      TNT_fn    Gelly_fn        diff
       BOND       2.000        5655    5161.365    5161.365       0.000
       ANGL       2.000        7674   11703.798   11703.798       0.000
       IMPR       0.000           0       0.000       0.000       0.000
       TORS       2.000        1968    2797.950    2797.950      -0.000
       TRIG       2.000           0       0.000       0.000       0.000
       PLAN       5.000         963   14444.369   14444.369       0.000
       CONT       5.000           0    9399.192       0.000    9399.192
       BCOR      10.000        5630  167873.806  167873.806      -0.000


 ============= Call Gelly geometry functions

   Switching torsion functional form to sinusoidal rather than split harmonic functional.
      if you want this not to happen then rerun with '-torsharm'
      Weight for sinusoidal torsion=     2.000
   BCOR function/gradient will be split between H and non-H atoms (as for any atom pair). Use '-bcorrel_all_in_hydrogens' to change this.

 ----------- Fix B-correlation restraints

 
 Checking for bonds that do not have a bcorrel restraint defined
 We will wipe all existing BCORrels if OCC=0.0 atom(s) is/are involved. This can be' switched off via the '-bcorrel_nowipe_zeroocc' flag.
 We will wipe all existing BCORrels if only non-hydrogen atom(s) with fixed B(iso) are involved. This can be switched off via the '-bcorrel_nowipe_fixb_nonhydrogens' flag.
 We will keep all existing BCORrels if hydrogen atom(s) with fixed B(iso) is/are involved. This can be switched off via the '-bcorrel_wipe_fixb_hydrogens' flag.
 No -bcorrel_missing_sigma found. So will set sigma to     2.5 Ang**2 for any bond with no existing BCORrel
 No -bcorrel_missing_sigma_hydrogens found. So will set sigma to     1.0 Ang**2 for any bond with no existing BCORrel involving hydrogens

 No active BCORels found involving OCC=0.0 atom(s)


 Have removed a total of      5630 BCORrels involving fixed-B (only) non-hydrogen atom(s)


 All bonds have a corresponding BCORrel - so no new added


 Have skipped a total of        25 bonds to setup BCORrels where both atoms have constant/fixed B(iso)


 ----------- Deactivate hydrogen-free planes if hydrogen atoms present

 A total of       0 planes have been deactivated because they are duplicated hydrogen-free and with-hydrogen forms.

 ----------- Handle peptide omega angle(s)

      Torsion angles split into peptide omega and other
         Number of peptide omega angles is       698
         Number of other torsion is             1270
      Peptide omega angles are monitored rather than directly restrained (PLANE used instead).

 ----------- Fix cis-PRO

 Now apply the fix to get correct restraints for cis PRO residues
   Have got      11 bonds records and      26 angles
   from GEOMETRY cards for the special PROC residue
   Weight for ideal-distance contact term=     4.000

 ----------- Prepare SSBOND/LINK for output PDB file


 ----------- Prepare for QM


 ----------- Prepare symmetry in utility distance restraints


 ############### Setting up Rhofit contacts (if needed)

 No ligand-(fixed protein) contact term will be used

 ############### Working out initial function and gradient


   MaxLik Scaling Cycle 0002
 
 Initial functional value = 3.999380E+05 rms gradient=      104.089
 X-ray contribution to function value = 2.240276E+05
 Geometry contribution (inc ncs)      = 1.759104E+05
 Geometry function summary
     term                           number     weight       rms        GooF      function     fn/numb
     ================================================================================================
     BOND bond lengths (angs)       5655       2.000      0.0136       0.676      5161.37       0.913
     ANGL bond angles (degs)        7674       2.000        1.47       0.873     11703.80       1.525
     TORS split-harmonic tors       1270       0.000       16.68       1.050         0.00       0.000
     SINTOR sinusoidal tors         1270       2.000       16.68                  2196.42       1.729
     PLAN general plane (angs)       963       5.000      0.0346       1.732     14444.37      14.999
     BCORrelations (angs^2)            0      10.000       0.000       0.000         0.00
     CONT (bad contacts)               0       5.000                                 0.00
     IDEAL(ideal-dist contact)      5937       4.000                             37912.60
     CHIRAL (gelly semiharmon)       740       5.000                                 8.07
     SIM similarity restraint for NCS and/or TARGET                             104483.81
     OCCSUM restraint on sum of occupanies                                           0.00       as none defined
     DISTAN utility restraints interatomic distance                                  0.00       as none defined
     UTILANGLE utility angle restraints                                              0.00       as none defined
     UTILTOR utility restraints on torsion angles                                    0.00       as none defined
     Peptide omega torsion angles                           7.50 degs - monitor only
 
 WARNING: Have |delta/sigma| deviations > 5.0 sigma. Number of outliers for each term: 
 WARNING:         2 bond angles.  Worst is     6.2 sigs   117.20 degs A|90:NE=CZ=NH2 (ARG)
 WARNING:        11 planes.       Worst is     7.0 sigs     0.14 Angs A|401:O7=C3=C6=O6 (CIT)
 WARNING:        13 idealD contacts. Worst    15.5 sigs     0.65 Angs B|401:C04(LIG)=S|407:O(HOH)

 Weighted rms      0.0136         1.465  (temporary rms bond and angle for BUSTER to pick up!)
 Weighted intra-residual rms(bond) for StdProtein (excluding H)      0.0140
 Weighted intra-residual rms(bond) for Buffer     (excluding H)      0.0273

 ############### Running initial check


 Initial Geometry Sanity Check

 Check for duplicate bonds: no duplicates found
 
 Check for duplicate or missing bond angles: all are OK
 Check for bond between an atom and itself not involving symmetry:  all are OK
 *** WARNING: There are 6 bad contacts where Delta/sigma is greater than      6.5
 *** WARNING: Looking at each one in turn to see whether it is likely to indicate a setup problem with geometry restraints.

        1 dist=   0.65 idealD=   3.66 sigma=   0.19 abs(Delta/sigma)=   15.53
          Between atoms   B|401:C04 (LIG)         and   S|407:O (HOH)           (C-O)
          occupancies=     0.40        1.00
 [check-023]
          Involves Water and non-Water atoms - so probably just badly placed
          water (but check).

        2 dist=   0.69 idealD=   3.58 sigma=   0.22 abs(Delta/sigma)=   13.19
          Between atoms   B|401:C10 (LIG)         and   S|485:O (HOH)           (C-O)
          occupancies=     0.40        1.00
 [check-023]
          Involves Water and non-Water atoms - so probably just badly placed
          water (but check).

        3 dist=   0.90 idealD=   3.58 sigma=   0.22 abs(Delta/sigma)=   12.27
          Between atoms   B|401:C09 (LIG)         and   S|485:O (HOH)           (C-O)
          occupancies=     0.40        1.00
 [check-023]
          Involves Water and non-Water atoms - so probably just badly placed
          water (but check).

        4 dist=   1.68 idealD=   3.66 sigma=   0.19 abs(Delta/sigma)=   10.21
          Between atoms   B|401:C02 (LIG)         and   S|407:O (HOH)           (C-O)
          occupancies=     0.40        1.00
 [check-023]
          Involves Water and non-Water atoms - so probably just badly placed
          water (but check).

        5 dist=   0.89 idealD=   2.92 sigma=   0.21 abs(Delta/sigma)=    9.59
          Between atoms   B|401:N05 (LIG)         and   S|407:O (HOH)           (N-O)
          occupancies=     0.40        1.00
 [check-023]
          Involves Water and non-Water atoms - so probably just badly placed
          water (but check).

        6 dist=   2.04 idealD=   3.58 sigma=   0.22 abs(Delta/sigma)=    6.88
          Between atoms   B|401:C06 (LIG)         and   S|407:O (HOH)           (C-O)
          occupancies=     0.40        1.00
 [check-023]
          Involves Water and non-Water atoms - so probably just badly placed
          water (but check).
 GEOMETRY CHECK OVERALL:  WARN


 ############### Creating geometry/screen report


 ############### Option "tao" - run Tao optimizer

 TAO: Toolkit for Advanced Optimization. Reference: 
     Munson, T., Sarich, J., Wild, S., Benson, S., McInnes, L.C. (2018)
     "TAO User Manual (Revision 3.9)", Mathematics and Computer Science
     Division, Argonne National Laboratory
     ANL/MCS-TM-322, http://www.mcs.anl.gov/petsc/
 
 Have found -keeppdb flag so will write out a .pdb file every 1000000 its during opt
 Will apply TAO optimization 
 Maximum number of iterations      100
 Convergence test on Grms       4.0000 (-glim)
 Limit on rms displacement to original position (rmsD2init): NONE test inactive (-dlim)

    analysing argument no 0 : -autoncs with j=0
    analysing argument no 1 : -hydrogens_input_occ2zero with j=0
    analysing argument no 2 : -hydrogens_output_occ2heavy with j=0
    analysing argument no 3 : -screen with j=0
    analysing argument no 4 : 100 with j=0
    analysing argument no 5 : -screen_sigma with j=0
    analysing argument no 6 : 3.0 with j=0
    analysing argument no 7 : -keeppdb with j=0
    analysing argument no 8 : 1000000 with j=0
    analysing argument no 9 : -taomonitor_verbose with j=0
    found argument starting with "-tao" : -taomonitor_verbose
    analysing argument no 10 : 2 with j=1
    found value after -tao/-options_/-viewer_ argument : "2"
 PETSC/TAO: setting option "-taomonitor_verbose" = "2"


   MaxLik Scaling Cycle 0003
   Iters     Tot_Funct    Xray_Funct    Geom_Funct          Grms     rmsD2init     maxD2init             R         Rfree           LLG       LLGfree     rms(bond)    rms(angle)       Opttag_H
   Iters             K   B_IMPF_FRAG        K_MISS   B_TEMP_MISS   K_IMPF_MISS   B_IMPF_MISS        K_SOLV   B_TEMP_SOLV   K_IMPF_SOLV   B_IMPF_SOLV        B_TEMP     B_TEMP_11     B_TEMP_12     B_TEMP_22     B_TEMP_13     B_TEMP_23     B_TEMP_33      K_WILSON      B_WILSON      FGCYCLES       Scatag_H
   Iters          BOND         ANGLE      IMPROPER       TORSION    SINTORSION      TRIGONAL        PLANAR  BCORRELATION       CONTACT     IDEALDIST        CHIRAL    SIMILARITY       OCCSSUM  UTILDISTANCE     UTILANGLE   UTILTORSION        RHOFIT        ADP-01        ADP-02        ADP-03        ADP-04        ADP-05        ADP-06        ADP-07        ADP-08        ADP-09        ADP-10      RHOFITPC            QM       Funtag_H
       0  3.999380E+05  2.240276E+05  1.759104E+05       100.407  0.000000E+00  0.000000E+00      0.187802      0.223064  0.000000E+00  0.000000E+00     0.0135555       1.46537       Opttag_D
       0       74.1908       1.57919       1.00000  0.000000E+00       1.00000  0.000000E+00      0.879445       78.3188       1.00000       28.0968  0.000000E+00       8.52886  0.000000E+00       8.52886  0.000000E+00  0.000000E+00      -17.0577       74.3233       2.27147        2       Scatag_D
       0       5161.37       11703.8  0.000000E+00  0.000000E+00       2196.42  0.000000E+00       14444.4  0.000000E+00  0.000000E+00       37912.6       8.06546  1.044838E+05 -1.000000E+10 -1.000000E+10 -1.000000E+10 -1.000000E+10 -1.000000E+10  0.000000E+00  0.000000E+00  0.000000E+00  0.000000E+00  0.000000E+00  0.000000E+00  0.000000E+00  0.000000E+00  0.000000E+00  0.000000E+00  0.000000E+00  0.000000E+00       Funtag_D
   Geometry Restraint Deviations SCREEN Information

 

 WARNING: have displacement of 17.82 Angs from initial (> 15.00 Angs)
          for variable number= 1605 (type X        ; returned function value as   1.705558E+010 )
          Involving atom S|407:O
 WARNING: clipping occured in        2 TLS group(s) with NCLIPS=    2780
 WARNING: clipping occured in        2 TLS group(s) with NCLIPS=    2780

 An atom has moved more than     1.50 A from the initial/last
 position - if this was an accepted trial point, probably need to update the
 non-bonded contact list.


   MaxLik Scaling Cycle 0004
 WARNING: clipping occured in        2 TLS group(s) with NCLIPS=    2301
 WARNING: clipping occured in        2 TLS group(s) with NCLIPS=    2301

 An atom has moved more than     1.50 A from the initial/last
 position - if this was an accepted trial point, probably need to update the
 non-bonded contact list.


   MaxLik Scaling Cycle 0005
 WARNING: clipping occured in        2 TLS group(s) with NCLIPS=     707
 WARNING: clipping occured in        2 TLS group(s) with NCLIPS=     707

   MaxLik Scaling Cycle 0006

   MaxLik Scaling Cycle 0007

   Wilson plot

   Statistics in resolution bins after refinement cycle 0001
```


|  |  |
| --- | --- |
|  | - Rxpct factors plot- Rxpct factors table- <Log-Lik Gain>- Ln Sigma A- Correlation Coefficients |


```
   Iters     Tot_Funct    Xray_Funct    Geom_Funct          Grms     rmsD2init     maxD2init             R         Rfree           LLG       LLGfree     rms(bond)    rms(angle)
   Iters             K   B_IMPF_FRAG        K_MISS   B_TEMP_MISS   K_IMPF_MISS   B_IMPF_MISS        K_SOLV   B_TEMP_SOLV   K_IMPF_SOLV   B_IMPF_SOLV        B_TEMP     B_TEMP_11     B_TEMP_12     B_TEMP_22     B_TEMP_13     B_TEMP_23     B_TEMP_33      K_WILSON      B_WILSON      FGCYCLES
   Iters          BOND         ANGLE      IMPROPER       TORSION    SINTORSION      TRIGONAL        PLANAR  BCORRELATION       CONTACT     IDEALDIST        CHIRAL    SIMILARITY       OCCSSUM  UTILDISTANCE     UTILANGLE   UTILTORSION        RHOFIT        ADP-01        ADP-02        ADP-03        ADP-04        ADP-05        ADP-06        ADP-07        ADP-08        ADP-09        ADP-10      RHOFITPC            QM
       1  3.989910E+05  2.243366E+05  1.746544E+05       251.645    0.00449729     0.0870780      0.187077      0.221634   -0.00154527    0.00138867     0.0135555       1.46537       Opttag_D
       1       75.2994       1.72646       1.00000  0.000000E+00       1.00000  0.000000E+00      0.885208       79.7922       1.00000       27.7185  0.000000E+00       7.85813  0.000000E+00       7.85813  0.000000E+00  0.000000E+00      -15.7163       74.3233       2.27147        4       Scatag_D
       1       5161.37       11703.8  0.000000E+00  0.000000E+00       2196.42  0.000000E+00       14444.4  0.000000E+00  0.000000E+00       36656.5       8.06546  1.044838E+05 -1.000000E+10 -1.000000E+10 -1.000000E+10 -1.000000E+10 -1.000000E+10  0.000000E+00  0.000000E+00  0.000000E+00  0.000000E+00  0.000000E+00  0.000000E+00  0.000000E+00  0.000000E+00  0.000000E+00  0.000000E+00  0.000000E+00  0.000000E+00       Funtag_D
   Geometry Restraint Deviations SCREEN Information

 

   MaxLik Scaling Cycle 0008

   Wilson plot

   Statistics in resolution bins after refinement cycle 0002
```


|  |  |
| --- | --- |
|  | - Rxpct factors plot- Rxpct factors table- <Log-Lik Gain>- Ln Sigma A- Correlation Coefficients |


```
   Iters     Tot_Funct    Xray_Funct    Geom_Funct          Grms     rmsD2init     maxD2init             R         Rfree           LLG       LLGfree     rms(bond)    rms(angle)
   Iters             K   B_IMPF_FRAG        K_MISS   B_TEMP_MISS   K_IMPF_MISS   B_IMPF_MISS        K_SOLV   B_TEMP_SOLV   K_IMPF_SOLV   B_IMPF_SOLV        B_TEMP     B_TEMP_11     B_TEMP_12     B_TEMP_22     B_TEMP_13     B_TEMP_23     B_TEMP_33      K_WILSON      B_WILSON      FGCYCLES
   Iters          BOND         ANGLE      IMPROPER       TORSION    SINTORSION      TRIGONAL        PLANAR  BCORRELATION       CONTACT     IDEALDIST        CHIRAL    SIMILARITY       OCCSSUM  UTILDISTANCE     UTILANGLE   UTILTORSION        RHOFIT        ADP-01        ADP-02        ADP-03        ADP-04        ADP-05        ADP-06        ADP-07        ADP-08        ADP-09        ADP-10      RHOFITPC            QM
       2  3.871720E+05  2.207525E+05  1.664196E+05       78.2486     0.0490165       1.01139      0.183004      0.218247     0.0163586     0.0133882     0.0135555       1.46537       Opttag_D
       2       75.2095       1.52174       1.00000  0.000000E+00       1.00000  0.000000E+00      0.899549       79.1013       1.00000       27.0314  0.000000E+00       8.46129  0.000000E+00       8.46129  0.000000E+00  0.000000E+00      -16.9226       74.3233       2.27147        1       Scatag_D
       2       5161.37       11703.8  0.000000E+00  0.000000E+00       2196.42  0.000000E+00       14444.4  0.000000E+00  0.000000E+00       28421.8       8.06546  1.044838E+05 -1.000000E+10 -1.000000E+10 -1.000000E+10 -1.000000E+10 -1.000000E+10  0.000000E+00  0.000000E+00  0.000000E+00  0.000000E+00  0.000000E+00  0.000000E+00  0.000000E+00  0.000000E+00  0.000000E+00  0.000000E+00  0.000000E+00  0.000000E+00       Funtag_D
   Geometry Restraint Deviations SCREEN Information

 
 WARNING: clipping occured in        1 TLS group(s) with NCLIPS=      13
 WARNING: clipping occured in        1 TLS group(s) with NCLIPS=      13

 An atom has moved more than     1.50 A from the initial/last
 position - if this was an accepted trial point, probably need to update the
 non-bonded contact list.

       3  3.858306E+05  2.207467E+05  1.650839E+05       127.896     0.0596514       1.33860      0.183856      0.219117     0.0163873    0.00759736     0.0135555       1.46537       Opttag_D
       3       73.4317       1.41894       1.00000  0.000000E+00       1.00000  0.000000E+00      0.900517       77.9550       1.00000       26.8129  0.000000E+00       8.52363  0.000000E+00       8.52363  0.000000E+00  0.000000E+00      -17.0473       74.3233       2.27147        2       Scatag_D
       3       5161.37       11703.8  0.000000E+00  0.000000E+00       2196.42  0.000000E+00       14444.4  0.000000E+00  0.000000E+00       27086.1       8.06546  1.044838E+05 -1.000000E+10 -1.000000E+10 -1.000000E+10 -1.000000E+10 -1.000000E+10  0.000000E+00  0.000000E+00  0.000000E+00  0.000000E+00  0.000000E+00  0.000000E+00  0.000000E+00  0.000000E+00  0.000000E+00  0.000000E+00  0.000000E+00  0.000000E+00       Funtag_D

 An atom has moved more than     1.50 A from the initial/last
 position - if this was an accepted trial point, probably need to update the
 non-bonded contact list.

       4  3.839815E+05  2.205781E+05  1.634034E+05       132.579     0.0783123       1.88392      0.182542      0.217067     0.0172297     0.0131274     0.0135555       1.46537       Opttag_D
       4       74.8267       1.54071       1.00000  0.000000E+00       1.00000  0.000000E+00      0.907555       80.0929       1.00000       26.6039  0.000000E+00       8.07751  0.000000E+00       8.07751  0.000000E+00  0.000000E+00      -16.1550       74.3233       2.27147        1       Scatag_D
       4       5161.37       11703.8  0.000000E+00  0.000000E+00       2196.42  0.000000E+00       14444.4  0.000000E+00  0.000000E+00       25405.6       8.06546  1.044838E+05 -1.000000E+10 -1.000000E+10 -1.000000E+10 -1.000000E+10 -1.000000E+10  0.000000E+00  0.000000E+00  0.000000E+00  0.000000E+00  0.000000E+00  0.000000E+00  0.000000E+00  0.000000E+00  0.000000E+00  0.000000E+00  0.000000E+00  0.000000E+00       Funtag_D

 An atom has moved more than     1.50 A from the initial/last
 position - need to update the non-bonded contact list.


 ############################################################################################################
 ###################################################### setting up contact terms
 ############################################################################################################

       5  3.824898E+05  2.198198E+05  1.626700E+05       62.1676     0.0889364       2.18827      0.181706      0.217170     0.0210174     0.0120029     0.0135555       1.46537       Opttag_D
       5       75.0635       1.50868       1.00000  0.000000E+00       1.00000  0.000000E+00      0.910682       80.4522       1.00000       26.6830  0.000000E+00       8.35409  0.000000E+00       8.35409  0.000000E+00  0.000000E+00      -16.7082       74.3233       2.27147        1       Scatag_D
       5       5161.37       11703.8  0.000000E+00  0.000000E+00       2196.42  0.000000E+00       14444.4  0.000000E+00  0.000000E+00       24672.2       8.06546  1.044838E+05 -1.000000E+10 -1.000000E+10 -1.000000E+10 -1.000000E+10 -1.000000E+10  0.000000E+00  0.000000E+00  0.000000E+00  0.000000E+00  0.000000E+00  0.000000E+00  0.000000E+00  0.000000E+00  0.000000E+00  0.000000E+00  0.000000E+00  0.000000E+00       Funtag_D
       6  3.806915E+05  2.191941E+05  1.614974E+05       51.0784      0.107194       2.74095      0.180748      0.217574     0.0241431    0.00867368     0.0135555       1.46537       Opttag_D
       6       76.0773       1.53897       1.00000  0.000000E+00       1.00000  0.000000E+00      0.916254       82.4665       1.00000       26.7648  0.000000E+00       8.75068  0.000000E+00       8.75068  0.000000E+00  0.000000E+00      -17.5014       74.3233       2.27147        1       Scatag_D
       6       5161.37       11703.8  0.000000E+00  0.000000E+00       2196.42  0.000000E+00       14444.4  0.000000E+00  0.000000E+00       23499.6       8.06546  1.044838E+05 -1.000000E+10 -1.000000E+10 -1.000000E+10 -1.000000E+10 -1.000000E+10  0.000000E+00  0.000000E+00  0.000000E+00  0.000000E+00  0.000000E+00  0.000000E+00  0.000000E+00  0.000000E+00  0.000000E+00  0.000000E+00  0.000000E+00  0.000000E+00       Funtag_D
       7  3.793400E+05  2.186348E+05  1.607052E+05       57.5614      0.120992       3.23016      0.180296      0.218324     0.0269371    0.00423090     0.0135555       1.46537       Opttag_D
       7       75.9262       1.50354       1.00000  0.000000E+00       1.00000  0.000000E+00      0.919146       83.2741       1.00000       26.6032  0.000000E+00       9.02987  0.000000E+00       9.02987  0.000000E+00  0.000000E+00      -18.0597       74.3233       2.27147        1       Scatag_D
       7       5161.37       11703.8  0.000000E+00  0.000000E+00       2196.42  0.000000E+00       14444.4  0.000000E+00  0.000000E+00       22707.4       8.06546  1.044838E+05 -1.000000E+10 -1.000000E+10 -1.000000E+10 -1.000000E+10 -1.000000E+10  0.000000E+00  0.000000E+00  0.000000E+00  0.000000E+00  0.000000E+00  0.000000E+00  0.000000E+00  0.000000E+00  0.000000E+00  0.000000E+00  0.000000E+00  0.000000E+00       Funtag_D

 An atom has moved more than     1.50 A from the initial/last
 position - if this was an accepted trial point, probably need to update the
 non-bonded contact list.

       8  3.782134E+05  2.179474E+05  1.602660E+05       41.2211      0.133191       3.65516      0.179712      0.218711     0.0303706    0.00127049     0.0135555       1.46537       Opttag_D
       8       75.5406       1.44767       1.00000  0.000000E+00       1.00000  0.000000E+00      0.920878       83.7483       1.00000       26.3909  0.000000E+00       9.17497  0.000000E+00       9.17497  0.000000E+00  0.000000E+00      -18.3499       74.3233       2.27147        1       Scatag_D
       8       5161.37       11703.8  0.000000E+00  0.000000E+00       2196.42  0.000000E+00       14444.4  0.000000E+00  0.000000E+00       22268.2       8.06546  1.044838E+05 -1.000000E+10 -1.000000E+10 -1.000000E+10 -1.000000E+10 -1.000000E+10  0.000000E+00  0.000000E+00  0.000000E+00  0.000000E+00  0.000000E+00  0.000000E+00  0.000000E+00  0.000000E+00  0.000000E+00  0.000000E+00  0.000000E+00  0.000000E+00       Funtag_D

 An atom has moved more than     1.50 A from the initial/last
 position - need to update the non-bonded contact list.


 ############################################################################################################
 ###################################################### setting up contact terms
 ############################################################################################################

       9  3.778019E+05  2.175945E+05  1.602075E+05       49.8012      0.143017       4.08842      0.179076      0.218994     0.0321337   -0.00454543     0.0135555       1.46537       Opttag_D
       9       75.7826       1.44799       1.00000  0.000000E+00       1.00000  0.000000E+00      0.922025       85.5303       1.00000       26.1553  0.000000E+00       9.22296  0.000000E+00       9.22296  0.000000E+00  0.000000E+00      -18.4459       74.3233       2.27147        1       Scatag_D
       9       5161.37       11703.8  0.000000E+00  0.000000E+00       2196.42  0.000000E+00       14444.4  0.000000E+00  0.000000E+00       22209.7       8.06546  1.044838E+05 -1.000000E+10 -1.000000E+10 -1.000000E+10 -1.000000E+10 -1.000000E+10  0.000000E+00  0.000000E+00  0.000000E+00  0.000000E+00  0.000000E+00  0.000000E+00  0.000000E+00  0.000000E+00  0.000000E+00  0.000000E+00  0.000000E+00  0.000000E+00       Funtag_D
      10  3.773277E+05  2.171457E+05  1.601820E+05       24.9954      0.144970       4.16671      0.178760      0.218414     0.0343753   -0.00190885     0.0135555       1.46537       Opttag_D
      10       75.5040       1.40739       1.00000  0.000000E+00       1.00000  0.000000E+00      0.921915       84.8595       1.00000       26.2336  0.000000E+00       9.22233  0.000000E+00       9.22233  0.000000E+00  0.000000E+00      -18.4447       74.3233       2.27147        1       Scatag_D
      10       5161.37       11703.8  0.000000E+00  0.000000E+00       2196.42  0.000000E+00       14444.4  0.000000E+00  0.000000E+00       22184.2       8.06546  1.044838E+05 -1.000000E+10 -1.000000E+10 -1.000000E+10 -1.000000E+10 -1.000000E+10  0.000000E+00  0.000000E+00  0.000000E+00  0.000000E+00  0.000000E+00  0.000000E+00  0.000000E+00  0.000000E+00  0.000000E+00  0.000000E+00  0.000000E+00  0.000000E+00       Funtag_D
      11  3.771280E+05  2.170017E+05  1.601263E+05       18.3818      0.145418       4.18791      0.178590      0.218375     0.0350947   -0.00199397     0.0135555       1.46537       Opttag_D
      11       75.4593       1.39754       1.00000  0.000000E+00       1.00000  0.000000E+00      0.921841       84.9108       1.00000       26.1559  0.000000E+00       9.24287  0.000000E+00       9.24287  0.000000E+00  0.000000E+00      -18.4857       74.3233       2.27147        1       Scatag_D
      11       5161.37       11703.8  0.000000E+00  0.000000E+00       2196.42  0.000000E+00       14444.4  0.000000E+00  0.000000E+00       22128.4       8.06546  1.044838E+05 -1.000000E+10 -1.000000E+10 -1.000000E+10 -1.000000E+10 -1.000000E+10  0.000000E+00  0.000000E+00  0.000000E+00  0.000000E+00  0.000000E+00  0.000000E+00  0.000000E+00  0.000000E+00  0.000000E+00  0.000000E+00  0.000000E+00  0.000000E+00       Funtag_D
      12  3.768811E+05  2.167892E+05  1.600919E+05       23.6048      0.150927       4.36023      0.178212      0.218260     0.0361562   -0.00324105     0.0135555       1.46537       Opttag_D
      12       75.7163       1.40484       1.00000  0.000000E+00       1.00000  0.000000E+00      0.922396       85.7548       1.00000       26.0829  0.000000E+00       9.26777  0.000000E+00       9.26777  0.000000E+00  0.000000E+00      -18.5355       74.3233       2.27147        1       Scatag_D
      12       5161.37       11703.8  0.000000E+00  0.000000E+00       2196.42  0.000000E+00       14444.4  0.000000E+00  0.000000E+00       22094.0       8.06546  1.044838E+05 -1.000000E+10 -1.000000E+10 -1.000000E+10 -1.000000E+10 -1.000000E+10  0.000000E+00  0.000000E+00  0.000000E+00  0.000000E+00  0.000000E+00  0.000000E+00  0.000000E+00  0.000000E+00  0.000000E+00  0.000000E+00  0.000000E+00  0.000000E+00       Funtag_D
      13  3.768165E+05  2.167296E+05  1.600869E+05       25.7724      0.154155       4.44778      0.178246      0.218773     0.0364540   -0.00623697     0.0135555       1.46537       Opttag_D
      13       75.5062       1.38830       1.00000  0.000000E+00       1.00000  0.000000E+00      0.922425       85.8100       1.00000       26.0408  0.000000E+00       9.36373  0.000000E+00       9.36373  0.000000E+00  0.000000E+00      -18.7275       74.3233       2.27147        2       Scatag_D
      13       5161.37       11703.8  0.000000E+00  0.000000E+00       2196.42  0.000000E+00       14444.4  0.000000E+00  0.000000E+00       22089.1       8.06546  1.044838E+05 -1.000000E+10 -1.000000E+10 -1.000000E+10 -1.000000E+10 -1.000000E+10  0.000000E+00  0.000000E+00  0.000000E+00  0.000000E+00  0.000000E+00  0.000000E+00  0.000000E+00  0.000000E+00  0.000000E+00  0.000000E+00  0.000000E+00  0.000000E+00       Funtag_D
      14  3.767257E+05  2.166518E+05  1.600739E+05       16.0734      0.157251       4.51910      0.178116      0.218882     0.0368427   -0.00737482     0.0135555       1.46537       Opttag_D
      14       75.6474       1.39310       1.00000  0.000000E+00       1.00000  0.000000E+00      0.922781       86.2162       1.00000       26.0452  0.000000E+00       9.38654  0.000000E+00       9.38654  0.000000E+00  0.000000E+00      -18.7731       74.3233       2.27147        1       Scatag_D
      14       5161.37       11703.8  0.000000E+00  0.000000E+00       2196.42  0.000000E+00       14444.4  0.000000E+00  0.000000E+00       22076.1       8.06546  1.044838E+05 -1.000000E+10 -1.000000E+10 -1.000000E+10 -1.000000E+10 -1.000000E+10  0.000000E+00  0.000000E+00  0.000000E+00  0.000000E+00  0.000000E+00  0.000000E+00  0.000000E+00  0.000000E+00  0.000000E+00  0.000000E+00  0.000000E+00  0.000000E+00       Funtag_D
      15  3.765738E+05  2.165469E+05  1.600269E+05       9.32354      0.164243       4.65972      0.178003      0.219209     0.0373666    -0.0101539     0.0135555       1.46537       Opttag_D
      15       75.7691       1.39677       1.00000  0.000000E+00       1.00000  0.000000E+00      0.923346       86.7949       1.00000       26.0689  0.000000E+00       9.39410  0.000000E+00       9.39410  0.000000E+00  0.000000E+00      -18.7882       74.3233       2.27147        1       Scatag_D
      15       5161.37       11703.8  0.000000E+00  0.000000E+00       2196.42  0.000000E+00       14444.4  0.000000E+00  0.000000E+00       22029.0       8.06546  1.044838E+05 -1.000000E+10 -1.000000E+10 -1.000000E+10 -1.000000E+10 -1.000000E+10  0.000000E+00  0.000000E+00  0.000000E+00  0.000000E+00  0.000000E+00  0.000000E+00  0.000000E+00  0.000000E+00  0.000000E+00  0.000000E+00  0.000000E+00  0.000000E+00       Funtag_D

 An atom has moved more than     1.50 A from the initial/last
 position - if this was an accepted trial point, probably need to update the
 non-bonded contact list.

      16  3.764904E+05  2.165055E+05  1.599850E+05       18.0221      0.169272       4.74395      0.177910      0.219438     0.0375736    -0.0112958     0.0135555       1.46537       Opttag_D
      16       75.9272       1.40772       1.00000  0.000000E+00       1.00000  0.000000E+00      0.923748       87.3941       1.00000       26.0520  0.000000E+00       9.36525  0.000000E+00       9.36525  0.000000E+00  0.000000E+00      -18.7305       74.3233       2.27147        1       Scatag_D
      16       5161.37       11703.8  0.000000E+00  0.000000E+00       2196.42  0.000000E+00       14444.4  0.000000E+00  0.000000E+00       21987.1       8.06546  1.044838E+05 -1.000000E+10 -1.000000E+10 -1.000000E+10 -1.000000E+10 -1.000000E+10  0.000000E+00  0.000000E+00  0.000000E+00  0.000000E+00  0.000000E+00  0.000000E+00  0.000000E+00  0.000000E+00  0.000000E+00  0.000000E+00  0.000000E+00  0.000000E+00       Funtag_D

 An atom has moved more than     1.50 A from the initial/last
 position - need to update the non-bonded contact list.


 ############################################################################################################
 ###################################################### setting up contact terms
 ############################################################################################################

      17  3.764076E+05  2.164505E+05  1.599571E+05       8.12396      0.171917       4.77209      0.177895      0.219476     0.0378482    -0.0115826     0.0135555       1.46537       Opttag_D
      17       75.7968       1.40045       1.00000  0.000000E+00       1.00000  0.000000E+00      0.923668       87.3410       1.00000       26.0227  0.000000E+00       9.29338  0.000000E+00       9.29338  0.000000E+00  0.000000E+00      -18.5868       74.3233       2.27147        1       Scatag_D
      17       5161.37       11703.8  0.000000E+00  0.000000E+00       2196.42  0.000000E+00       14444.4  0.000000E+00  0.000000E+00       21959.3       8.06546  1.044838E+05 -1.000000E+10 -1.000000E+10 -1.000000E+10 -1.000000E+10 -1.000000E+10  0.000000E+00  0.000000E+00  0.000000E+00  0.000000E+00  0.000000E+00  0.000000E+00  0.000000E+00  0.000000E+00  0.000000E+00  0.000000E+00  0.000000E+00  0.000000E+00       Funtag_D
      18  3.763435E+05  2.164160E+05  1.599275E+05       6.91352      0.173306       4.76177      0.177891      0.219434     0.0380203    -0.0115035     0.0135555       1.46537       Opttag_D
      18       75.6617       1.39482       1.00000  0.000000E+00       1.00000  0.000000E+00      0.923511       87.1963       1.00000       25.9430  0.000000E+00       9.21760  0.000000E+00       9.21760  0.000000E+00  0.000000E+00      -18.4352       74.3233       2.27147        1       Scatag_D
      18       5161.37       11703.8  0.000000E+00  0.000000E+00       2196.42  0.000000E+00       14444.4  0.000000E+00  0.000000E+00       21929.7       8.06546  1.044838E+05 -1.000000E+10 -1.000000E+10 -1.000000E+10 -1.000000E+10 -1.000000E+10  0.000000E+00  0.000000E+00  0.000000E+00  0.000000E+00  0.000000E+00  0.000000E+00  0.000000E+00  0.000000E+00  0.000000E+00  0.000000E+00  0.000000E+00  0.000000E+00       Funtag_D
      19  3.762980E+05  2.163891E+05  1.599088E+05       8.48314      0.174901       4.74591      0.177854      0.219526     0.0381548    -0.0116483     0.0135555       1.46537       Opttag_D
      19       75.6560       1.39512       1.00000  0.000000E+00       1.00000  0.000000E+00      0.923452       87.2633       1.00000       25.8748  0.000000E+00       9.17594  0.000000E+00       9.17594  0.000000E+00  0.000000E+00      -18.3519       74.3233       2.27147        1       Scatag_D
      19       5161.37       11703.8  0.000000E+00  0.000000E+00       2196.42  0.000000E+00       14444.4  0.000000E+00  0.000000E+00       21911.0       8.06546  1.044838E+05 -1.000000E+10 -1.000000E+10 -1.000000E+10 -1.000000E+10 -1.000000E+10  0.000000E+00  0.000000E+00  0.000000E+00  0.000000E+00  0.000000E+00  0.000000E+00  0.000000E+00  0.000000E+00  0.000000E+00  0.000000E+00  0.000000E+00  0.000000E+00       Funtag_D
      20  3.762554E+05  2.163484E+05  1.599070E+05       6.83403      0.175904       4.72742      0.177773      0.219494     0.0383584    -0.0113830     0.0135555       1.46537       Opttag_D
      20       75.7077       1.39792       1.00000  0.000000E+00       1.00000  0.000000E+00      0.923430       87.3779       1.00000       25.8241  0.000000E+00       9.15208  0.000000E+00       9.15208  0.000000E+00  0.000000E+00      -18.3042       74.3233       2.27147        1       Scatag_D
      20       5161.37       11703.8  0.000000E+00  0.000000E+00       2196.42  0.000000E+00       14444.4  0.000000E+00  0.000000E+00       21909.2       8.06546  1.044838E+05 -1.000000E+10 -1.000000E+10 -1.000000E+10 -1.000000E+10 -1.000000E+10  0.000000E+00  0.000000E+00  0.000000E+00  0.000000E+00  0.000000E+00  0.000000E+00  0.000000E+00  0.000000E+00  0.000000E+00  0.000000E+00  0.000000E+00  0.000000E+00       Funtag_D
      21  3.762197E+05  2.163017E+05  1.599180E+05       12.5106      0.176825       4.69845      0.177707      0.219692     0.0385916    -0.0126086     0.0135555       1.46537       Opttag_D
      21       75.7428       1.39832       1.00000  0.000000E+00       1.00000  0.000000E+00      0.923400       87.5431       1.00000       25.7465  0.000000E+00       9.17476  0.000000E+00       9.17476  0.000000E+00  0.000000E+00      -18.3495       74.3233       2.27147        1       Scatag_D
      21       5161.37       11703.8  0.000000E+00  0.000000E+00       2196.42  0.000000E+00       14444.4  0.000000E+00  0.000000E+00       21920.2       8.06546  1.044838E+05 -1.000000E+10 -1.000000E+10 -1.000000E+10 -1.000000E+10 -1.000000E+10  0.000000E+00  0.000000E+00  0.000000E+00  0.000000E+00  0.000000E+00  0.000000E+00  0.000000E+00  0.000000E+00  0.000000E+00  0.000000E+00  0.000000E+00  0.000000E+00       Funtag_D
      22  3.761970E+05  2.162732E+05  1.599238E+05       11.8019      0.178171       4.68351      0.177636      0.219633     0.0387340    -0.0122964     0.0135555       1.46537       Opttag_D
      22       75.8048       1.40222       1.00000  0.000000E+00       1.00000  0.000000E+00      0.923454       87.7437       1.00000       25.7226  0.000000E+00       9.15659  0.000000E+00       9.15659  0.000000E+00  0.000000E+00      -18.3132       74.3233       2.27147        1       Scatag_D
      22       5161.37       11703.8  0.000000E+00  0.000000E+00       2196.42  0.000000E+00       14444.4  0.000000E+00  0.000000E+00       21926.0       8.06546  1.044838E+05 -1.000000E+10 -1.000000E+10 -1.000000E+10 -1.000000E+10 -1.000000E+10  0.000000E+00  0.000000E+00  0.000000E+00  0.000000E+00  0.000000E+00  0.000000E+00  0.000000E+00  0.000000E+00  0.000000E+00  0.000000E+00  0.000000E+00  0.000000E+00       Funtag_D
      23  3.761791E+05  2.162569E+05  1.599221E+05       6.35660      0.178527       4.67615      0.177626      0.219687     0.0388151    -0.0126284     0.0135555       1.46537       Opttag_D
      23       75.7823       1.40007       1.00000  0.000000E+00       1.00000  0.000000E+00      0.923455       87.7595       1.00000       25.7266  0.000000E+00       9.15982  0.000000E+00       9.15982  0.000000E+00  0.000000E+00      -18.3196       74.3233       2.27147        1       Scatag_D
      23       5161.37       11703.8  0.000000E+00  0.000000E+00       2196.42  0.000000E+00       14444.4  0.000000E+00  0.000000E+00       21924.3       8.06546  1.044838E+05 -1.000000E+10 -1.000000E+10 -1.000000E+10 -1.000000E+10 -1.000000E+10  0.000000E+00  0.000000E+00  0.000000E+00  0.000000E+00  0.000000E+00  0.000000E+00  0.000000E+00  0.000000E+00  0.000000E+00  0.000000E+00  0.000000E+00  0.000000E+00       Funtag_D
      24  3.761508E+05  2.162402E+05  1.599107E+05       3.86340      0.180440       4.65105      0.177640      0.219889     0.0388989    -0.0137067     0.0135555       1.46537       Opttag_D
      24       75.7153       1.39654       1.00000  0.000000E+00       1.00000  0.000000E+00      0.923476       87.8355       1.00000       25.7332  0.000000E+00       9.15190  0.000000E+00       9.15190  0.000000E+00  0.000000E+00      -18.3038       74.3233       2.27147        1       Scatag_D
      24       5161.37       11703.8  0.000000E+00  0.000000E+00       2196.42  0.000000E+00       14444.4  0.000000E+00  0.000000E+00       21912.8       8.06546  1.044838E+05 -1.000000E+10 -1.000000E+10 -1.000000E+10 -1.000000E+10 -1.000000E+10  0.000000E+00  0.000000E+00  0.000000E+00  0.000000E+00  0.000000E+00  0.000000E+00  0.000000E+00  0.000000E+00  0.000000E+00  0.000000E+00  0.000000E+00  0.000000E+00       Funtag_D

   MaxLik Scaling Cycle 0033

   Wilson plot

   Statistics in resolution bins after refinement cycle 0003
```


|  |  |
| --- | --- |
|  | - Rxpct factors plot- Rxpct factors table- <Log-Lik Gain>- Ln Sigma A- Correlation Coefficients |


```
   Iters     Tot_Funct    Xray_Funct    Geom_Funct          Grms     rmsD2init     maxD2init             R         Rfree           LLG       LLGfree     rms(bond)    rms(angle)
   Iters             K   B_IMPF_FRAG        K_MISS   B_TEMP_MISS   K_IMPF_MISS   B_IMPF_MISS        K_SOLV   B_TEMP_SOLV   K_IMPF_SOLV   B_IMPF_SOLV        B_TEMP     B_TEMP_11     B_TEMP_12     B_TEMP_22     B_TEMP_13     B_TEMP_23     B_TEMP_33      K_WILSON      B_WILSON      FGCYCLES
   Iters          BOND         ANGLE      IMPROPER       TORSION    SINTORSION      TRIGONAL        PLANAR  BCORRELATION       CONTACT     IDEALDIST        CHIRAL    SIMILARITY       OCCSSUM  UTILDISTANCE     UTILANGLE   UTILTORSION        RHOFIT        ADP-01        ADP-02        ADP-03        ADP-04        ADP-05        ADP-06        ADP-07        ADP-08        ADP-09        ADP-10      RHOFITPC            QM
      25  3.761340E+05  2.162363E+05  1.598977E+05       5.56570      0.182002       4.65815      0.177634      0.219945     0.0389183    -0.0141921     0.0135555       1.46537       Opttag_D
      25       75.7342       1.39803       1.00000  0.000000E+00       1.00000  0.000000E+00      0.923540       87.9673       1.00000       25.7617  0.000000E+00       9.15247  0.000000E+00       9.15247  0.000000E+00  0.000000E+00      -18.3049       74.3233       2.27147        1       Scatag_D
      25       5161.37       11703.8  0.000000E+00  0.000000E+00       2196.42  0.000000E+00       14444.4  0.000000E+00  0.000000E+00       21899.9       8.06546  1.044838E+05 -1.000000E+10 -1.000000E+10 -1.000000E+10 -1.000000E+10 -1.000000E+10  0.000000E+00  0.000000E+00  0.000000E+00  0.000000E+00  0.000000E+00  0.000000E+00  0.000000E+00  0.000000E+00  0.000000E+00  0.000000E+00  0.000000E+00  0.000000E+00       Funtag_D
   Geometry Restraint Deviations SCREEN Information

 
 No -bcorrel_zeroocchydrogens_factor flag found - keeping default: will set B for OCC=0 hydrogens to    1.200 times heavy-atom B


 TLS information. Total number of groups:     2


 Group   Natoms    FixRB   FixTLS   Group Tag    Group Spec
     1     2770      Y        N     tls1    { A|* }
     2     2770      Y        N     tls2    { B|* }
 
NOTE BUSTER_TLS_SET tls1 { A|* }
NOTE BUSTER_TLS_T   tls1     -5.14088E-02  1.73416E-02 -8.39463E-02 -8.98636E-03  2.71161E-02 -1.44550E-02
NOTE BUSTER_TLS_L   tls1      5.75955E-01  6.03476E-01  7.20382E-02  4.29501E-01 -2.01688E-01 -7.44442E-02
NOTE BUSTER_TLS_S   tls1      2.27569E-01 -1.40005E-01  1.08103E-01 -1.53415E-01 -6.25968E-02 -9.66768E-02 -5.90336E-02  8.69448E-04
NOTE BUSTER_TLS_O   tls1     -2.34016E+01 -8.37990E+01  1.17779E+01
NOTE BUSTER_TLS_FIX tls1 RB
 
NOTE BUSTER_TLS_SET tls2 { B|* }
NOTE BUSTER_TLS_T   tls2     -6.53873E-02 -2.61952E-02 -1.61508E-02  1.87470E-02  4.10405E-02  3.19663E-03
NOTE BUSTER_TLS_L   tls2      3.60326E-01  5.33835E-01  2.67053E-01  2.19756E-01 -1.98637E-01 -2.16915E-01
NOTE BUSTER_TLS_S   tls2     -1.51205E-02  2.91585E-02 -3.20999E-02 -6.51209E-03  1.30308E-01  8.58995E-03 -4.97491E-02  3.47416E-02
NOTE BUSTER_TLS_O   tls2      2.21707E+01 -7.20650E+01  3.37962E+00
NOTE BUSTER_TLS_FIX tls2 RB

 For final position, Geometry function summary
     term                           number     weight       rms        GooF      function     fn/numb
     ================================================================================================
     BOND bond lengths (angs)       5655       2.000      0.0136       0.676      5161.37       0.913
     ANGL bond angles (degs)        7674       2.000        1.47       0.873     11703.80       1.525
     TORS split-harmonic tors       1270       0.000       16.68       1.050         0.00       0.000
     SINTOR sinusoidal tors         1270       2.000       16.68                  2196.42       1.729
     PLAN general plane (angs)       963       5.000      0.0346       1.732     14444.37      14.999
     BCORrelations (angs^2)            0      10.000       0.000       0.000         0.00
     CONT (bad contacts)               0       5.000                                 0.00
     IDEAL(ideal-dist contact)      5339       4.000                             21899.88
     CHIRAL (gelly semiharmon)       740       5.000                                 8.07
     SIM similarity restraint for NCS and/or TARGET                             104483.81
     OCCSUM restraint on sum of occupanies                                           0.00       as none defined
     DISTAN utility restraints interatomic distance                                  0.00       as none defined
     UTILANGLE utility angle restraints                                              0.00       as none defined
     UTILTOR utility restraints on torsion angles                                    0.00       as none defined
     Peptide omega torsion angles                           7.50 degs - monitor only
 
 WARNING: Have |delta/sigma| deviations > 5.0 sigma. Number of outliers for each term: 
 WARNING:         2 bond angles.  Worst is     6.2 sigs   117.20 degs A|90:NE=CZ=NH2 (ARG)
 WARNING:        11 planes.       Worst is     7.0 sigs     0.14 Angs A|401:O7=C3=C6=O6 (CIT)
 WARNING:         1 idealD contacts. Worst     5.1 sigs     2.53 Angs A|91:CB=O (ASP)

 Weighted rms      0.0136         1.465  (temporary rms bond and angle for BUSTER to pick up!)
 Weighted intra-residual rms(bond) for StdProtein (excluding H)      0.0140
 Weighted intra-residual rms(bond) for Buffer     (excluding H)      0.0273
 Outputing geometry progress graph in plotmtv format to file:Geom_Res.mtv

 ############### Running final geometry/screen analysis (screen_final.txt)

 s/r gelly normal termination
```

Statistics and scale factors vs. cycle number
```

|  |  |  |
| --- | --- | --- |
|  | - Rxpct factors- <Log-Lik Gain>- GEOM Residuals- Ln Sigma_A_ | - Scale Factors- B Factors- Imperfection Factors |

```
   Final Geometry Information


---


### 2. Final results

Statistics and scale factors vs. cycle number
```

|  |  |  |
| --- | --- | --- |
|  | - Rxpct factors- <Log-Lik Gain>- GEOM Residuals- Ln Sigma_A_ | - Scale Factors- B Factors- Imperfection Factors |

```
   Average Figure of Merit
   Structure Factor Amplitudes


 # normal termination

  Program stopped.
```

closesok message: time spent in tree = 426.88 (user) 10.55 (sys)

---

# Normal termination

```
 Resources usage summary:
     Time spent in client ...................   426.89 (user)       10.57 (system)
     Time spent in scripts (    27 calls) ...     1.55 (user)        0.14 (system)
     Time spent in server ...................     0.00 (user)        0.01 (system)

     Max resident memory used (kb) .......... 441068
     Number of page faults .................. 0 (major)    227747 (minor)
     Number of context switches ............. 361398 (niced)    2444 (other)
```
